# Supplementary figures and images for: Synergetic effect of non-complementary 5’ AT-rich sequences on the development of a multiplex TaqMan real-time PCR for specific and robust detection of Clavibacter michiganensis and C. michiganensis subsp. nebraskensis
Source: PLoS One. 2019 Jul 11;14(7):e0218530. doi: 10.1371/journal.pone.0218530 (PMC6622472; doi:10.1371/journal.pone.0218530)

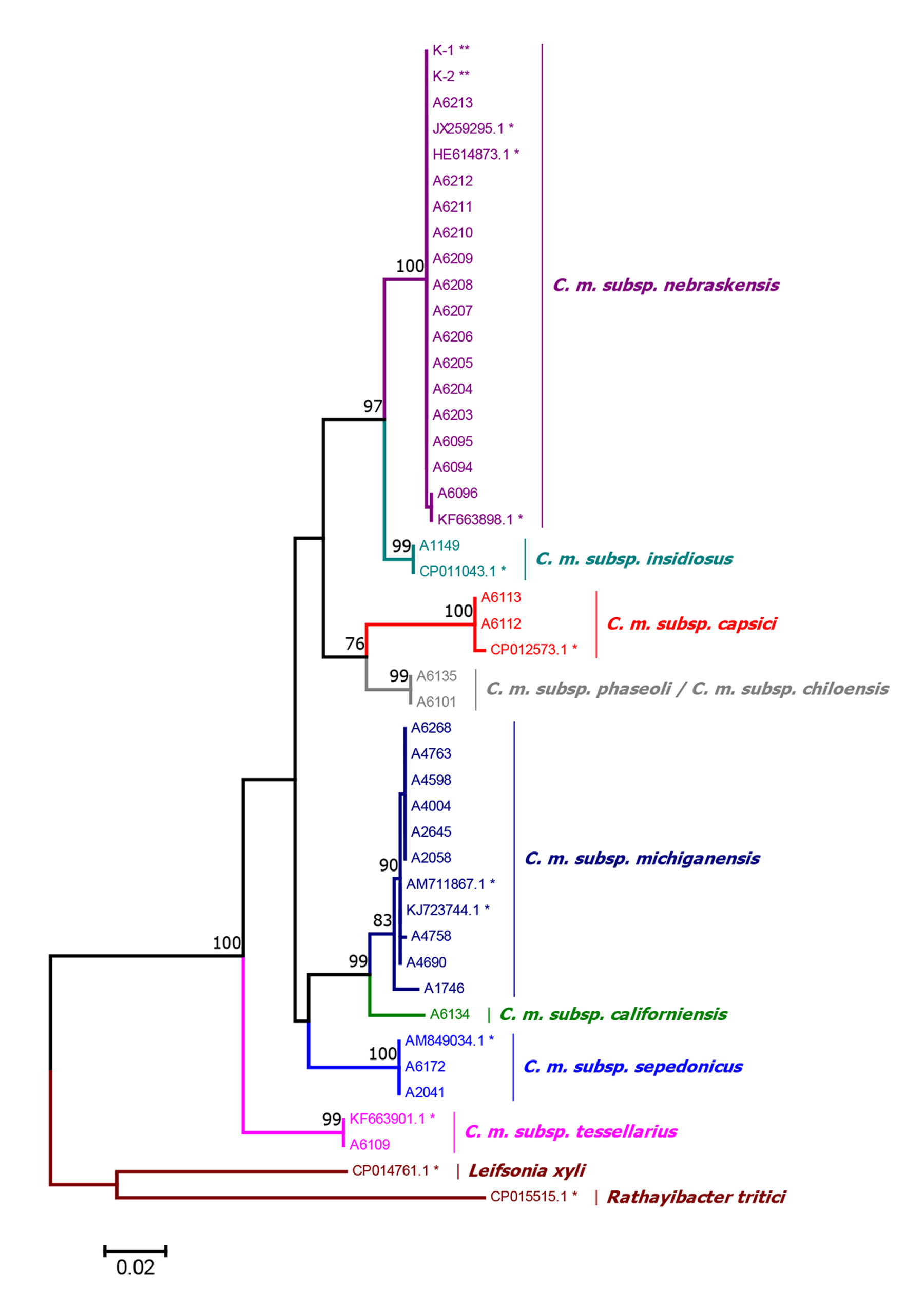

Supplement: S1 Fig — The evolutionary distances were computed using a General Time Reversible model with gamma distribution (GTR + G). Forty-three partial sequences based on dnaA gene of Clavibacter strains were rooted with 2 closest taxa Leifsonia xyli and Rathayibacter tritici, served as an outgroup. A 1,000 replicates were performed to calculate the bootstrap value which is shown over the branches; only bootstrap values greater than 70% are presented. (JPG) [file pone.0218530.s001.jpg]

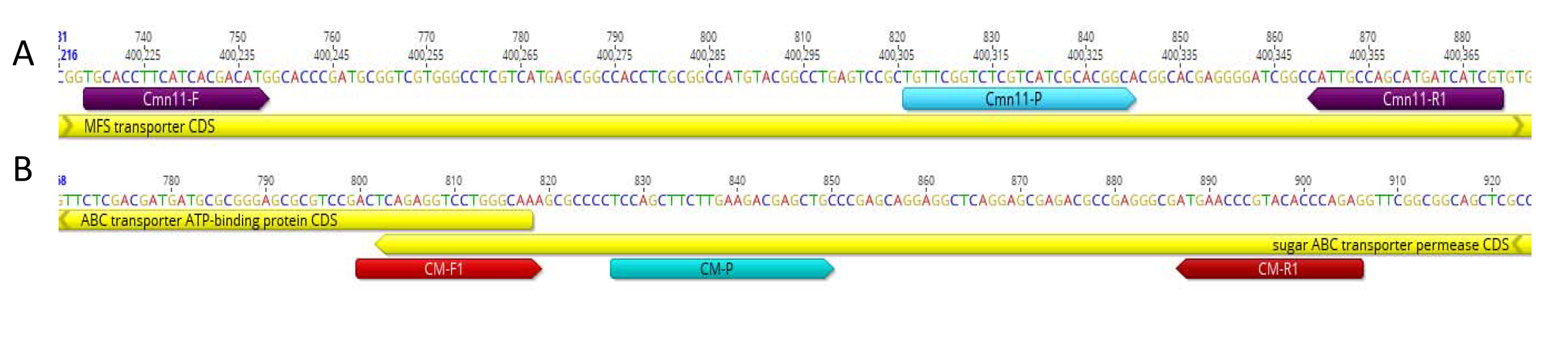

Supplement: S2 Fig — A. Primers and probe (Cmn11-F/R/P) targeting the MFS transporter gene specific for C. m. subsp. nebraskensis. B. Primers and probe (CM-F/R/P) targeting the sugar ABC transporter permease and ABC transporter ATP-binding genes for specific detection of C. michiganensis species. In both cases, the probe is located in the inner part of the amplicon, between the sense and anti-sense primers. (JPG) [file pone.0218530.s002.jpg]

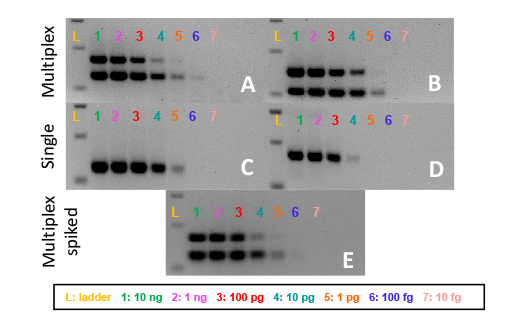

Supplement: S3 Fig — (A) and (B) Multiplex reactions performed for detection of subspecies within the C. michiganensis and C. m. subsp. nebraskensis with/without 5’ AT-rich sequences, respectively; (C). Single reactions targeting MSF gene of C. m. subsp. nebraskensis; (D) Single reactions targeting the sugar ABC transporter permease and ABC transporter ATP-binding genes for specific detection of C. michiganensis subspecies; (E) 1 μl host corn DNA was added in each reaction of ten-fold serially diluted sensitivity assay for simultaneous detection of C. m. subsp. nebraskensis and other C. michiganensis subspecies. All the experiments were conducted the same day using genomic DNA from C. m. subsp. nebraskensis. A molecular-weight size marker of 100 bp from BioLabs was used as a standard reference to determine the product size. All PCR products were electrophoresed in a 3% agarose gel. (TIF) [file pone.0218530.s003.tif]
